# FMCS: a novel algorithm for the multiple MCS problem

**DOI:** 10.1186/1758-2946-5-S1-O6

**Published:** 2013-03-22

**Authors:** Andrew Dalke, Janna Hastings

**Affiliations:** 1 Andrew Dalke Scientific AB, Göteborg, Sweden; 2 Cheminformatics and Metabolism, EBI, Hinxton, UK

## 

Clustering and classification of large-scale chemical data are essential for navigation, analysis and knowledge discovery in a wide variety of chemical application domains. The maximum common structure (MCS) for a group of compounds is an important element of such classification, providing insight into activity patterns and enabling scaffold alignment for a more consistent 2D depiction. Most modern, exact MCS implementations use back-tracking [1] or clique detection [2], and handle the multiple MCS problem by recursive reduction to successive pairwise maximal common substructure searches [3]. We present fmcs, which implements a novel multiple MCS algorithm based on subgraph enumeration and subgraph isomorphism testing [4,5] and with algorithm improvements and heuristics which make it competitive to the standard methods. MCS performance evaluation is very sensitive to the test set, so we have developed several reference benchmarks based on ChEMBL-13, including randomly selected pairs of structures, and randomly selected structures with their k = 2, k = 10, and k = 100 nearest neighbors. We use these benchmarks to compare fmcs to SMSD [6] and Indigo's scaffold detector [7]. Most differences are due to chemistry perception and timeout errors. The fmcs performance, written in Python using the RDKit C++ toolkit [8], is currently between 0.3x and 1.2x the performance of the Indigo implementation in C++. We also cross-validated the fmcs algorithm with the manually curated ChEBI structure ontology classification [9] and characterized the differences. We identified limitations with fmcs, such as with tautomer perception and structural classes that fmcs cannot handle, and problems with ChEBI, such as misclassifications and classifications that are not, structurally speaking, strictly hierarchical.

## References

[B1] McGregor JJ Backtrack Search Algorithms and the Maximal Common Subgraph Problem Software-Practice and Experience 1982 12 23 34 10.1002/spe.4380120103

[B2] Raymond JW Willett P Maximum common subgraph isomorphism algorithms for the matching of chemical structures J Comp Aid Mol Des 2002 16 521 533 10.1023/A:1021271615909 12510884

[B3] Hariharan R Janakiraman A Nilakantan R Singh B Varghese S Landrum G Schuffenhauer A MultiMCS: A Fast Algorithm for the Maximum Common Substructure Problem on Multiple Molecules J Chem Inf Mod 2011 51 788 806 10.1021/ci100297y 21446748

[B4] Varkony T Shiloach Y Smith D Computer-Assisted Examination of Chemical Compounds for Structural Similarities J Chem Inf Comp Sci 1979 19 104 111 10.1021/ci60018a014

[B5] Takahashi Y Satoh Y Suzuki H Sasaki S Recognition of Largest Structural Fragment among a Variety of Chemical Structures Anal Sci 1987 3 23 28 10.2116/analsci.3.23

[B6] Rahman SA Holliday GL Schrader R Thornton JM Small Molecule Subgraph Detector (SMSD) Toolkit J Cheminf 2009 1 12 10.1186/1758-2946-1-12 PMC2820491 20298518

[B7] Indigo cheminformatics library http://ggasoftware.com/opensource/indigo/

[B8] RDKit cheminformatics library http://rdkit.org/

[B9] Degtyarenko K de Matos P Ennis M Hastings J Zbinden M McNaught A Alcántara R Darsow M Guedj M Ashburner M ChEBI: a database and ontology for chemical entities of biological interest Nucleic Acids Res 2008 36 D344 D350 17932057 10.1093/nar/gkm791 PMC2238832

